# A unified superatomic-molecule theory for local aromaticity in π-conjugated systems

**DOI:** 10.1093/nsr/nwac216

**Published:** 2022-10-14

**Authors:** Dan Li, Jinlong Yang, Longjiu Cheng

**Affiliations:** Key Laboratory of Structure and Functional Regulation of Hybrid Materials (Ministry of Education), Department of Chemistry, Anhui University, Hefei 230601, China; Hefei National Laboratory for Physical Sciences at the Microscale, University of Science and Technology of China, Hefei 230026, China; Key Laboratory of Structure and Functional Regulation of Hybrid Materials (Ministry of Education), Department of Chemistry, Anhui University, Hefei 230601, China

**Keywords:** aromaticity, superatom, superatomic molecule, chemical bond theory, multicenter bonding, electron delocalization

## Abstract

Aromaticity is one of the most important concepts in chemistry. However, there is still no unified chemical insight for various systems with conjugated sp^2^ carbon. Herein, we proposed a superatomic-molecule theory to build a generalized electron rule for polycyclic conjugated hydrocarbons, fullerenes and 2D periodic materials. Taking benzenoid units as 2D superatoms, polycyclic conjugated hydrocarbons and C_60_ can be seen as superatomic molecules consisting of bonded superatoms, resulting in local aromaticity. In superatomic molecules, π electrons are not totally delocalized, but localized in a single superatom forming superatomic lone pairs or shared by two atoms forming a superatomic bond, mimicking rules in classical valence bond theory. Moreover, two 2D superatomic crystals (C_18_H_6_ and C_54_H_18_) are predicted to have fairly large band gaps (∼1.8 eV), although the π electrons are conjugated and delocalized. The proposed superatomic-molecule theory provides generalized chemical insights into the nature of local aromaticity, which can be qualitatively evaluated by the chemical intuition given by superatomic Lewis structures.

## Teaser

A unified superatomic-molecule theory is proposed as a generalized language for aromaticity in p-conjugated systems: polycyclic conjugated hydrocarbons, fullerenes and 2D periodic materials.

## INTRODUCTION

The term ‘aromaticity’ was first introduced to refer to a group of molecules with particular properties by Hofmann in 1856 [1] and now it is considered as one of the most important concepts in organic chemistry. Aromaticity was initially related either to some typical properties or to specific 2D organic compounds, such as monocyclic planar conjugate hydrocarbons and their ions with (4*n* + 2)π electrons, polycyclic conjugated hydrocarbons (PCHs) built of fused benzene rings and polycyclic conjugated carbocyclic hydrocarbons based on non-benzenoid systems [2–4]. Today, the term has been further extended to a large number of heterocyclic compounds [5–9], 3D compounds, such as sandwich compounds [10,11], fullerenes [12,13], boron hydrides [14], boron clusters [15–17] and all-metal clusters [18–22], as well as bridged 2D–3D aromatic compounds [23].

In spite of many studies devoted to this subject, aromaticity is still a rather fuzzy concept. To understand the origin of aromaticity, a number of models are given using valence bond (VB) method (Kekulé structure), molecular orbital (MO) theory and graph theory [3]. There are also several criteria for aromaticity, such as the Hückel 4*n* + 2 rule [24], Clar's π-sextet (6*n*) rule [25], Möbius 4*n* rule [26], spherical 2(*n* + 1)^2^ rule [13], geometric indicators [27], electron delocalization measure [28,29], nuclear independent chemical shifts (NICS) value [30] and ring currents [31,32].

In concept, the nature of aromaticity is stabilization resulting from electron delocalization and aromatic rules for polycyclic conjugated hydrocarbons (PCHs), fullerenes and graphenes should be the same. However, due to the complexity and diversity, there is still no unified language for the aromaticity of molecules, clusters and 2D materials. NICS and ring currents are easy and efficient criteria for aromaticity, which have achieved great success in various systems. Chemical insights of the aromaticity are clear for simple monocyclic (Hückel 4*n* + 2 rule) and spherical [2(*n* + 1)^2^ rule] molecules. However, for the complex polycyclic systems, chemical insights behind the numerical rules and criterion values are still not so clear.

The jellium model [33] is widely used to describe the delocalized electrons in metallic clusters. All the nuclei and atomic innermost electrons of the cluster are taken as a positively charged entirety and provide a jellium-like potential field, where valence electrons move freely. Thus, the cluster behaves as a superatom with electronic shells |1S^2^|1P^6^|1D^10^2S^2^|1F^14^2P^6^|…, associated with magic numbers 2, 8, 20, 40…, in good agreement with the peaks viewed in the mass spectra of sodium clusters [33]. Superatom theory has achieved great successes in both bare and ligand-protected metallic clusters [34–39]. Later, we found that valence electrons in metallic clusters were not totally delocalized [40,41]. A non-spherical metallic cluster comprises two equivalent blocks, each following the electronic characteristic of a superatom. Similar to classical molecules, two superatoms share electron pairs and form a superatomic molecule with an electronic closed shell [42,43]. Superatom theory gives reasonable chemical insights into the electronic and geometric structures of metallic clusters.

Herein, based on the concept of the superatom, we proposed a 2D superatomic-molecule theory for π-conjugated systems, which gives reasonable chemical insights into the local aromaticity of PCHs, fullerenes and 2D periodic materials.

## RESULTS AND DISCUSSION

### 2D superatom model

**Figure 1.**
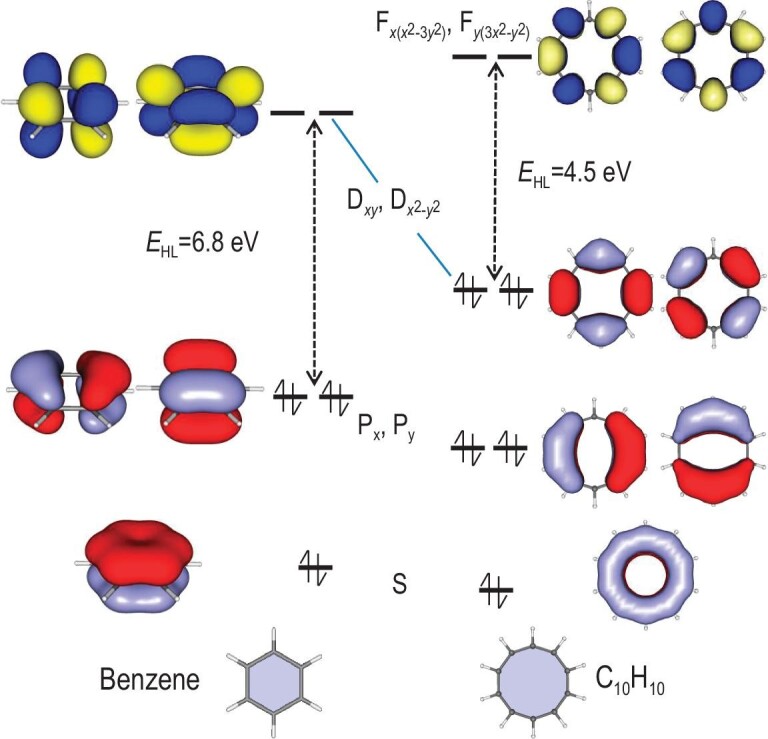
Superatom model of monocyclic C*_n_*H*_n_*. Canonical MO diagrams of the π orbitals of benzene and monocyclic C_10_H_10_ at the B3LYP/6–31G* level of theory. *E*_HL_ gives the HOMO–LUMO energy gap.

Similar to the cases in metallic clusters, π electrons in monocyclic C*_n_*H*_n_* molecules are conjugated and delocalized, which could be described with a 2D version of the jellium model. The π electrons are delocalized only over the C*_n_* ring, so principal quantum numbers of the superatomic orbitals are restricted to one, whereas angular quantum numbers are unrestricted. However, the π MOs stretch only in the *xy*-plane, and for any angular quantum number, the maximum degeneracy is two (see Supplementary Fig. S1 in the Supplementary information). Without consideration of the π symmetry, the appropriate Aufbau rule of this 2D jellium model is |S^2^|P^4^|D^4^|F^4^|… (capital letters are used to distinguish from that of atomic shells), associated with magic numbers 2, 6, 10, 14…, satisfying the Hückel 4*n* + 2 rule. Figure 1 gives the canonical MO diagrams of π electrons of benzene and monocyclic C_10_H_10_. Projection of the first MO can be seen as a superatomic S orbital. The second MO is doubly degenerate and can be seen as superatomic P*_x_* and P*_y_* orbitals. Similarly, the third and fourth are D*_xy_*―D*_x_*_2__–_*_y_*_2_ and F*_x_*_(_*_x_*_2__–3_*_y_*_2)_―F*_y_*_(3_*_x_*_2__–_*_y_*_2)_ orbitals, respectively. Besides the satisfaction of the sp^2^ σ-bonding framework of carbon, benzene also has fulfilled π superatomic orbitals (S^2^P^4^D^0^) with a very large energy gap (*E*_HL_ = 6.8 eV) between the highest occupied molecular orbital (HOMO) and the lowest unoccupied molecular orbital (LUMO). Thus, benzene can be seen as a six-electron 2D superatom (S^2^P^4^) in both geometric and electronic shell closure, which is a 2D analog of Ne (s^2^p^6^), and is symbolized as ^◊^Ne in this work. The monocyclic C_10_H_10_ can also be seen as a closed-shell superatom (S^2^P^4^D^4^F^0^) but the σ-bonding frameworks are strained.

### Superatomic-molecule model for PCHs

In theories of both aromaticity and superatoms, only the global character of electron delocalization is concerned and it is difficult to give reasonable chemical insights for large/complex systems. As we know, the electronic structure of a complex molecule can be described by VB theory in a simple and straightforward way via Lewis structures, where each atom reaches the electronic shell of the noble gas atom (eight-electron or octet rule) via sharing electron pairs with other atoms (chemical bonds). In a certain sense, the octet rule is the most important base of chemistry, which gives us the chemical sense.

**Figure 2.**
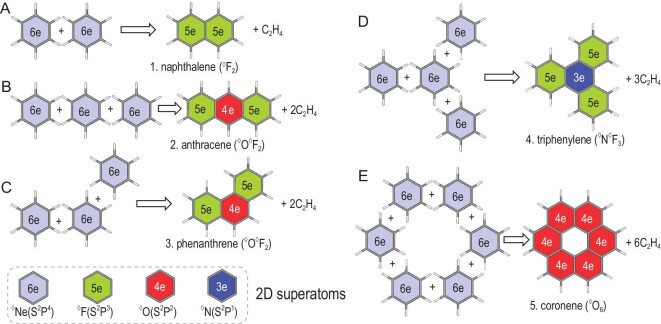
Illustrations for the construction of superatomic molecules of (A) **1**, naphthalene, (B) **2**, anthracene, (C) **3**, phenanthrene, (D) **4**, triphenylene and (E) **5**, coronene.

In terms of PCHs, although the π electrons are conjugated, local character should be concerned and then the whole π system can be seen as a 2D superatomic molecule consisting of bonded superatoms. Each 2D superatom can be open-shell, but the whole system has a molecule-like closed shell. As illustrated in Fig. 2A, naphthalene (**1**) is composed of two edge-fused benzenes, where each benzenoid unit has five electrons. The 5e 2D superatom (S^2^P^3^) is only one electron less than benzene (^◊^Ne) and is symbolized as ^◊^F. Thus, naphthalene (**1**) could be seen as a di-superatomic molecule (^◊^F_2_). Similarly, when one benzenoid unit is fused with two other ones, it is a 4e 2D superatom (S^2^P^2^) symbolized as ^◊^O. Anthracene (**2**) and phenanthrene (**3**) are tri-superatomic molecules, which can be seen as linear and bent ^◊^O^◊^F_2_, respectively. The central ^◊^O is SP-hybridized in **2** (Fig. 2B), whereas it is SP^2^-hybridized in **3** (Fig. 2C). When one benzenoid unit is fused with three others, it can be seen as a ^◊^N superatom (S^2^P^1^) in SP^2^ hybridization and thus triphenylene (**4**) is a superatomic ^◊^N^◊^F_3_ (Fig. 2D). Coronene (**5**) is composed of six benzenoid units fused in a cyclic manner and can be seen as a superatomic cyclic ^◊^O_6_, whereas the central benzenoid unit is just a hole (Fig. 2E). As we know, the maximum coordination number of a p-block element is four (octet rule of Ne). However, to meet the sextet rule of ^◊^Ne, the maximum coordination number of a 2D superatom is only three. It should be noted that although 2D superatoms have the same valance states as corresponding elements, the bonding patterns can be very different, where corresponding molecules might be unstable (such as O_6_).

**Figure 3.**
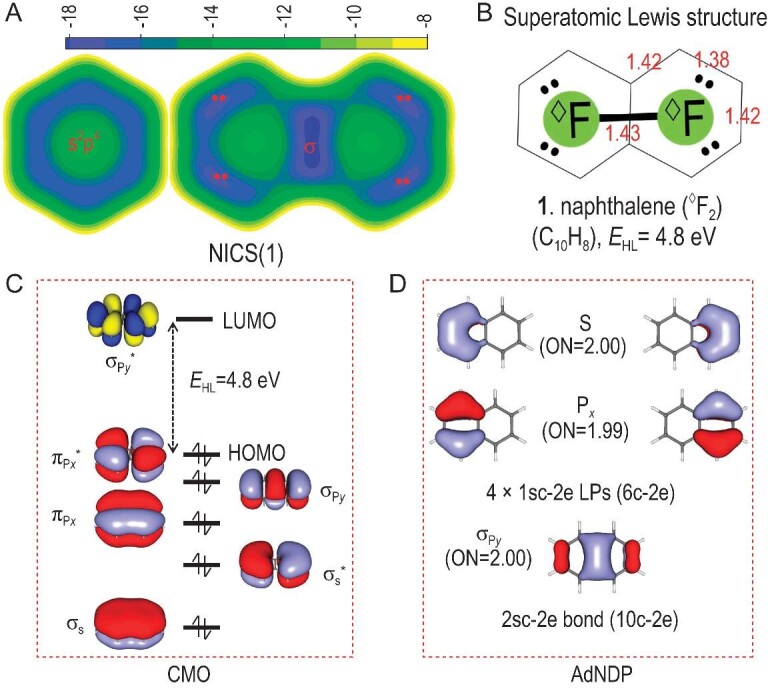
Superatomic-molecule presentation of naphthalene. (A) NICS(1) contour planes (ppm) above the molecular plane of 1.0 Å, (B) superatomic Lewis structures, (C) canonical π MO diagrams and (D) chemical bonding analysis of AdNDP of naphthalene (**1**). NICS(1) contour planes of benzene are also plotted for comparison. ON gives the occupancy numbers (|e|). The C–C bond lengths are given in Å (red).

To verify such a superatomic-molecule model in naphthalene (**1**), Fig. 3A compares the NICS(1) contour planes between benzene and **1**. It is clear that benzene shows uniform aromaticity, whereas the NICS(1) values of **1** exhibit the obvious local feature of superatomic ^◊^F_2_. A superatomic Lewis structure of **1** is given in Fig. 3B for clearer chemical insights. The length of the C–C bond also agrees well with the superatomic Lewis structure. From the symmetry of canonical π MO diagrams, electronic configurations of naphthalene can be seen as superatomic (σ_s_)^2^(σ*_s_)^2^(π_p_*_x_*)^2^(σ_p_*_y_*)^2^(π*_p_*_x_*)^2^(σ*_p_*_y_*)^0^ (Fig. 1C). Both bonding and anti-bonding orbitals composed of superatomic S and P*_x_* orbitals are occupied, suggesting superatomic LPs. The σ_py_ bonding orbital suggests a superatomic σ bond. The HOMO–LUMO gap between π*_px_ and σ*_py_ is fairly large (*E*_HL_ = 4.8 eV). To further verify the superatomic Lewis structure, Fig. 1D plots the localized orbitals of Adaptive Natural Density Partitioning (AdNDP) [44] analysis for the π frameworks. As expected, there are two six-center two-electron (6c–2e) π bonds localized in each superatom, which is regarded as one-supercenter two-electron (1sc–2e) superatomic LPs in this study. Similarly, the 10c–2e π orbital delocalized over two superatoms is defined as a 2sc–2e superatomic σ bond. With two superatomic LPs and one superatomic σ bond, each ^◊^F superatom satisfies the sextet rule of ^◊^Ne and thus a molecule-like closed shell is satisfied. The AdNDP bonding framework gives clear evidence for the suggested superatomic Lewis structure in both the orbital symmetry and occupancy numbers (ONs) of the bonds (ON = 1.99–2.00 |e| closed to the idealized value 2.00 |e|).

**Figure 4.**
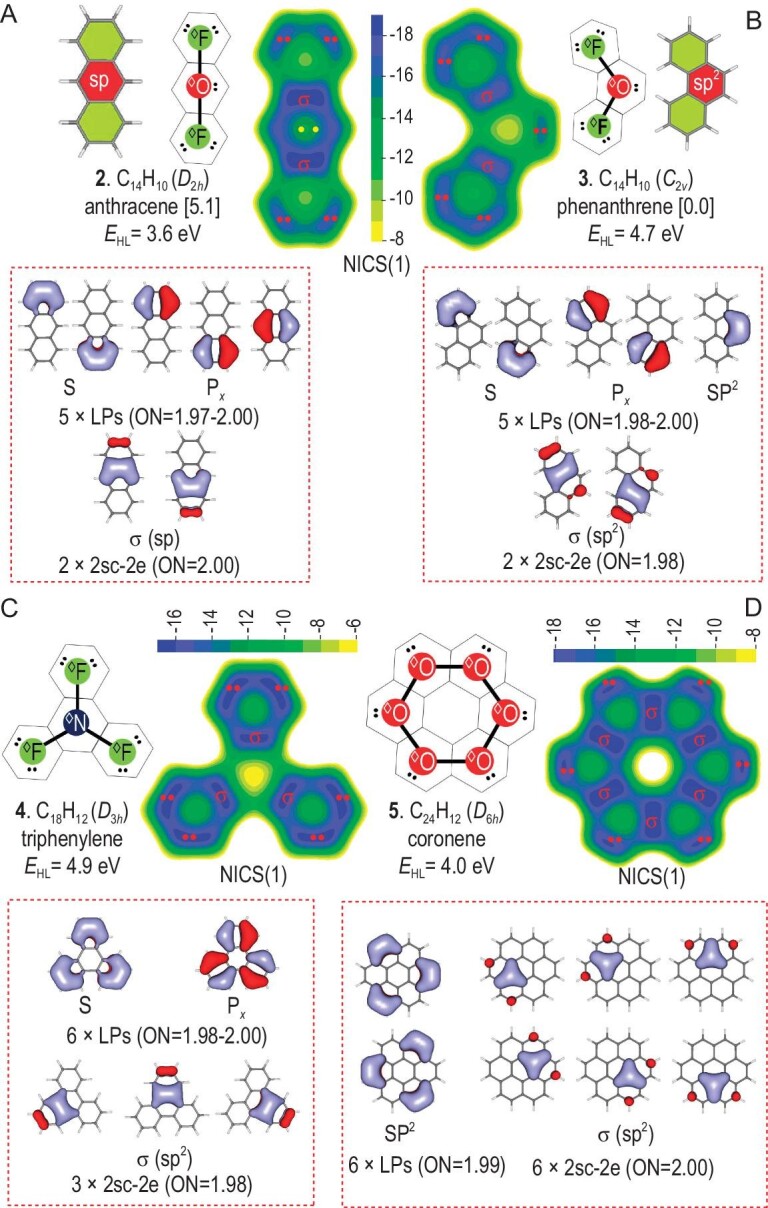
Comparison of superatomic Lewis structures, NICS(1) contour planes and AdNDP bonding frameworks. (A) **2**, anthracene (linear ^◊^O^◊^F_2_), (B) **3**, phenanthrene (bent ^◊^O^◊^F_2_), (C) **4**, triphenylene (^◊^N^◊^F_3_) and (D) **5**, coronene (^◊^O_6_). Enclosed are the relative energies in kcal/mol. ON gives the occupancy numbers (|e|).

Figure 4A and B compares the superatomic Lewis structures, NICS(1) contour planes and AdNDP bonding frameworks of anthracene (**2**) and phenanthrene (**3**), which can be seen as linear and bent superatomic ^◊^O^◊^F_2_, respectively. Canonical π MO diagrams of the tri-superatomic molecules are too complicated to give direct evidence for the suggested superatomic Lewis structures, whereas AdNDP bonding frameworks are straightforward with very high occupancy numbers (ON = 1.97–2.00 |e|). Two superatomic LPs of the terminal superatoms in **2** and **3** are similar to those in naphthalene (**1**), suggesting the same bonding patterns (^◊^F). However, superatomic LP of the central ^◊^O in **2** (SP-hybridized, pure P*_x_*) is totally different from that in **3** (SP^2^-hybridized), which are good analogies of those in classical VB theory from orbital symmetries. In addition, the types of superatomic LPs are responsible for the gap of NICS values between the central superatoms. P*_x_*-type LPs are more delocalized, leading to greater ring currents and more negative NICS values. However, more negative NICS values in the ^◊^O superatom of **2** does not mean higher aromaticity [45]. It is well known that **3** is more stable than **2** [46], which can be explained by our superatomic-molecule model. Similar to the ‘valence layer electron mutually exclusive theory’ in classical VB theory, SP^2^-hybridized ^◊^O is more favored than the SP-hybridized one (less repulsion between electron pairs).

As shown in Fig. 4C, the superatomic Lewis structure, NICS(1) contour plane and AdNDP bonding framework show straightforwardly that triphenylene (**4**, C_18_H_12_) is a tetra-superatomic ^◊^N^◊^F_3_. There are four linear isomers of **4** as tetra-superatomic ^◊^O_2_^◊^F_2_ as a result of different hybridization of ^◊^O superatoms, where SP^2^ hybridization is also more favored in energy (Supplementary Fig. S2). Similar to fatty saturated hydrocarbons with formula C*_n_*H_2_*_n_*_+2_, it can be easily concluded that the superatomic formula of such a chain-like *n*-superatomic molecule is ^◊^O*_n_*_–__2_^◊^F_2_ with 4*n* + 2 π electrons (C_4_*_n_*_+2_H_2_*_n_*_+4_), satisfying the Hückel 4*n* + 2 rule.

If superatoms are bonded in a cyclic manner, the number of π electrons will break the 4*n* + 2 rule. Figure 4D plots the superatomic Lewis structure, NICS(1) contour plane and AdNDP orbitals of coronene (**5**, C_24_H_12_), which is aromatic but has 4*n* π electrons. Although there are seven benzenoid units, coronene is composed of only six superatoms in a cyclic manner (^◊^O_6_), where the central benzenoid unit does not satisfy the sextet rule of ^◊^Ne and is just a hole (non-aromatic) as clearly shown in the NICS(1) contour plane. AdNDP analysis gives clear evidence for this inference, where each superatom is SP^2^-hybridized (two superatomic σ bonds and one superatomic LP).

The superatomic-molecule model can be easily extended to larger PCHs. Supplementary Fig. S3 gives an example of C_48_H_24_, which is a superatomic molecule bonded by coronene with six outer superatoms (^◊^N_6_^◊^F_6_). The bonding pattern of the central seven benzenoid units in C_48_H_24_ is the same as that of coronene. Supplementary Fig. S4 shows another three examples of local aromaticity, where C_20_H_12_, C_28_H_14_ and C_30_H_16_ can be seen as unions of two naphthalenes (^◊^F_2_ dimer), two anthracenes (^◊^O^◊^F_2_ dimer) and three naphthalenes (^◊^F_2_ trimer), respectively. Due to the failure in satisfying the sextet rule of ^◊^Ne, the connected benzenoid units are non-aromatic holes.

### Superatomic-molecule model in fullerenes

**Figure 5.**
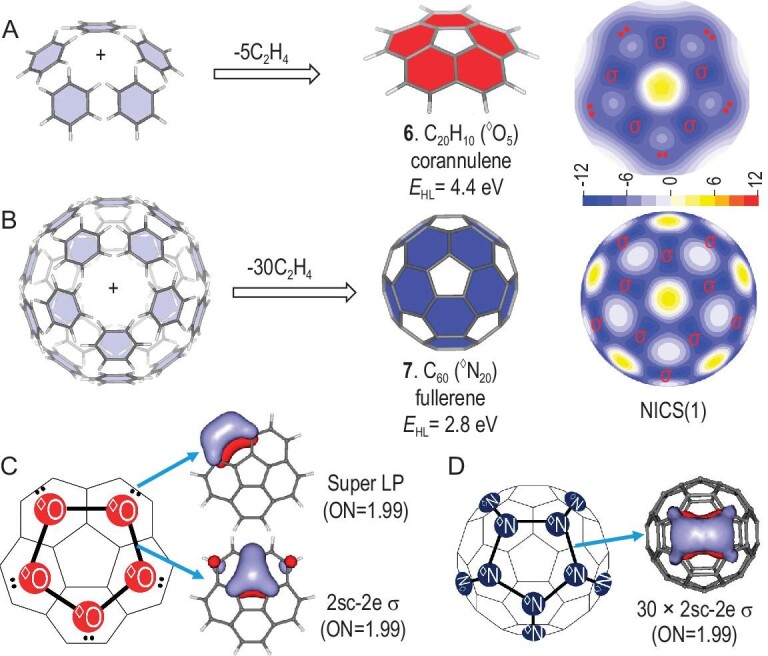
Superatomic-molecule illustrations for corannulene (^◊^O_5_) and C_60_ (^◊^N_20_). Growth patterns and NICS(1) contour planes (ppm) of (A) corannulene (**6**, C_20_H_10_) and (B) fullerene (**7**, C_60_). Superatomic Lewis structures and AdNDP localized orbitals of (C) corannulene (**6**) and (D) C_60_ (**7**).

Systems containing both five- and six-membered rings are also applicable by the superatomic-molecule model. Corannulene (**6**, C_20_H_10_) is a well-known aromatic molecule with a C_5_ ring surrounded by five C_6_ rings. As shown in Fig. 5A, it can be seen as a superatomic cyclic ^◊^O_5_, with the same bonding pattern as coronene (cyclic ^◊^O_6_). The central C_5_ unit is also a hole (non-aromatic), with even slightly positive NICS(1) values at the ring center.

C_60_ (**7**) is composed of 12 five- and 20 six-membered rings, which is of the highest aromaticity/stability among fullerenes from the perspective of delocalization energy and geometric factor [12]. However, the aromatic rule of C_60_ is still a challenge, which does not satisfy the 2(*n* + 1)^2^ rule of spherical aromaticity and was suggested to be globally non-aromatic due to the fact that there are no obvious ring currents and the NICS value at the cage center is very small [13]. The magic stability of C_60_ can be explained by our superatomic-molecule model, which can be seen as a superatomic ^◊^N_20_ cage with high local aromaticity (Fig. 5B). Corannulene (**6**) is a fragment of C_60_ in a geometric structure and it has similar superatomic-molecule bonding patterns. Interestingly, the local character of the NICS(1) contour plane of C_60_ is also very similar to that of corannulene (**6**). Such a superatomic-molecule model was also verified by the AdNDP localized orbitals with nearly idealized ONs (Fig. 5C and D). Therefore, the magic stability of C_60_ results from both electronic and geometric shell closure, where both 60 sp^2^ carbon atoms and 20 SP^2^-hybridized ^◊^N superatoms match the *I*_h_ symmetry. The superatomic-molecule model makes a bridge to the local aromaticity between PCHs and C_60_, and the aromaticity of other fullerenes can also be explained via similar strategies.

### Superatomic-crystal model in 2D materials

**Figure 6.**
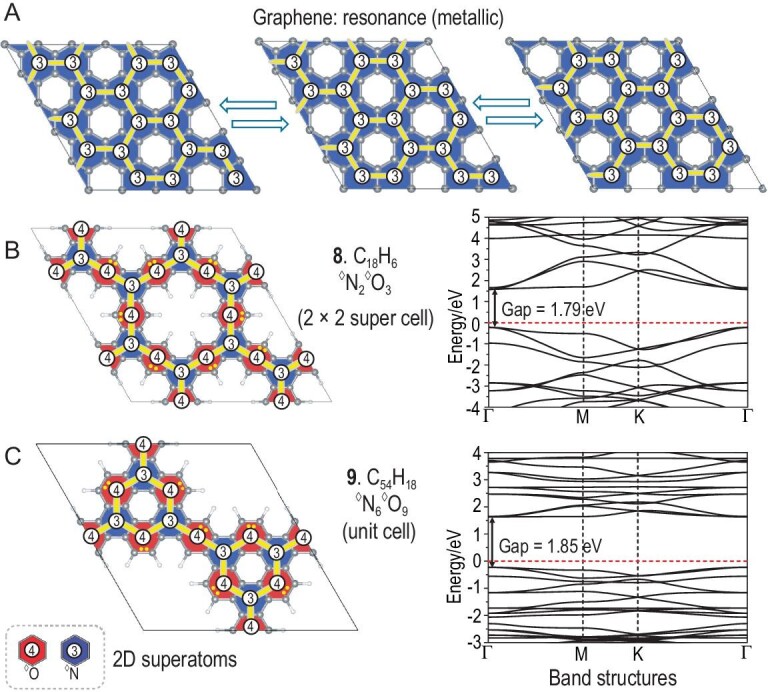
Superatomic-crystal model for 2D periodic metals. (A) Three resonant superatomic Lewis structures of graphene. Superatomic Lewis structures and band structures (PBE) of 2D superatomic crystals of (B) C_18_H_6_ (^◊^N_2_^◊^O_3_) and (C) C_54_H_18_ (^◊^N_6_^◊^O_9_). The Fermi level is set at zero.

For periodic systems, the electronic structure of graphene can also be described as a superatomic Lewis structure. Taking coronene (**5**, C_24_H_12_) as a building block, as shown in Fig. 6A, graphene can be seen as a superatomic crystal consisting of SP^2^-hybridized ^◊^N. However, there are three resonant superatomic Lewis structures with different locations of the holes, resulting in the total delocalization of π electrons and metallic properties.

In concept, in a superatomic crystal without resonance, π electrons are localized either in one superatom (superatomic LP) or between two superatoms (superatomic bonds), and thus there should be a reasonable band gap similar to the cases in covalent crystals. To verify this inference, taking anthracene (**2**) and triphenylene (**4**) as building blocks, we construct a C_18_H_6_ 2D periodic material (**8**, Fig. 6B). Details of the construction, superatomic-molecule growth pattern and analysis of electronic structures can be found in Supplementary Fig. S5. Based on our model, this material is a superatomic crystal (^◊^N_2_^◊^O_3_) in which each ^◊^O is SP-hybridized. As expected, such a superatomic crystal has a fairly large band gap (1.79 eV), although the π electrons are conjugated.

Similarly, as plotted in Fig. 6C, another 2D periodic material C_54_H_18_ (**9**) is constructed by using anthracene (**2**) and coronene (**5**) as building blocks. Details of the construction, **s**uperatomic-molecule growth pattern and analysis of electronic structures can be found in Supplementary Fig. S6. This superatomic crystal can be seen as ^◊^N_6_^◊^O_9_, with six SP^2^- and three SP-hybridized ^◊^O in one unit cell. **8** and **9** have the same chemical formula (C_3_H). However, due to more SP^2^-hybridized ^◊^O, **9** has a larger band gap (1.85 eV) and is lower in energy than **8** by ∼9.36 kcal/mol (per ^◊^N_2_^◊^O_3_ unit). Interestingly, the energy difference between SP^2^- and SP-hybridized ^◊^O is 4.7 kcal/mol in superatomic crystals of **8** and **9**, close to the value in superatomic molecules of anthracene and phenanthrene (5.1 kcal/mol).

Conjugated π electrons in 2D periodic materials are verified to follow the same rule as in PCHs and fullerenes. The band gap is a key parameter for some functional applications (such as electronic and optical devices) of 2D periodic materials. The superatomic-crystal theory may provide a theoretical foundation to modulate band gaps of graphene-like materials.

## DISCUSSION

It has been shown that NICS(1) contour planes are consistent with AdNDP bonding in local aromaticity, satisfying the superatomic-molecule theory. The current density is another popular criterion for aromaticity. As shown in Supplementary Fig. S8, there are diatropic ring currents in all the aromatic molecules, indicating the global characters of the conjugated π electrons. However, it is difficult to find local characters directly from the ring currents. In coronene (**5**), corannulene (**6**) and fullerene (**7**), superatomic-molecule theory suggested non-aromatic holes verified by both NICS(1) contour planes and AdNDP bonding. However, there are clear paratropic ring currents around the suggested non-aromatic holes (Supplementary Fig. S9), suggesting that they are anti-aromatic [47]. We conjecture that such disagreement is due to the misunderstanding of the ring currents. We think that, in locally aromatic molecules, regions between paratropic and diatropic ring currents are aromatic, and regions within paratropic ring currents are non-aromatic. To further verify such a supposition, Supplementary Fig. S10A compares the NICS(1) contour plane and ring currents of C_48_H_24_ (^◊^O_1_**_2_**), which has a large non-aromatic hole based on a superatomic-molecule model. As expected, there are clear diatropic and paratropic ring currents at the outer and inner borders of the aromatic region, respectively. C_156_H_60_ (^◊^N_12_^◊^O_30_) is an example with both large and small non-aromatic holes, where regions within paratropic ring currents are also non-aromatic according to the NICS(1) contour plane (Supplementary Fig. S10B). C_90_H_30_ is another example with a large hole but cannot be properly described by a superatomic Lewis structure, which has largely negative NICS values in whole molecular regions and has diatropic ring currents in both the outer and inner borders of the molecule, suggesting global aromaticity (Supplementary Fig. S10C). However, more negative NICS values and stronger ring currents do not always mean higher stability of electronic structures. Globally aromatic C_90_H_30_ has a obviously lower HOMO–LUMO energy gap (1.63 eV) than those of locally aromatic C_48_H_24_ (3.56 eV) and C_156_H_60_ (3.06 eV).

In globally aromatic molecules, conjugated π electrons are delocalized and fully occupy the superatomic molecular-orbital [Hückel 4*n* + 2 rule or spherical 2(*n* + 1)^2^ rule], resulting in closed-shell electronic structures and high stability. Direct pictures of global aromaticity can be given by the current density method (the higher the degree of electron delocalization, the stronger the global ring currents). The NICS value arises from ring currents, which gives a quantitative measurement for aromaticity (the more negative the NICS values, the higher the aromaticity). As common aromatic criteria, NICS and current density have achieved great success in indicating global aromaticity for a variety of monocyclic and cage molecules.

In polycyclic systems, to achieve a closed-shell electronic structure of superatomic molecules, π electrons are localized in a single superatom forming superatomic LPs or are shared by two atoms forming a superatomic bond. The partly delocalized superatomic LPs and bonds break the global delocalization of π electrons, leading to local aromaticity. NICS and current density are related to the degree of globally electronic delocalization and thus are weakened in superatomic molecules. In this situation, NICS values and ring currents do not always match the relative stability or amount of aromaticity. For example, SP^2^-hybridized ^◊^O is lower in energy, whereas SP-hybridized ^◊^O has more negative NICS values due to the fact that P*_x_*-type superatomic LP is more delocalized. Superatomic-molecule theory does not give quantitative measurement for aromaticity, but chemical insights behind the local characters of NICS(1) contour planes and paratropic/diatropic ring currents can be reasonably understood by superatomic Lewis structures. Atomic Lewis structures help us to build a chemical intuition for the stability and property of molecules. Similarly, the stability or aromaticity of π-conjugated systems can be qualitatively evaluated by the chemical intuition from superatomic Lewis structures. It should be noted that both atomic and superatomic Lewis structures are responsible for the stability of superatomic molecules. For example, in the four ^◊^O_2_^◊^F_2_ isomers (Supplementary Fig. S2), chrysene has two SP^2^-hybridized ^◊^O and is the most stable, while tetracene has two SP-hybridized ^◊^O and is the highest in energy. However, benzophenanthrene (two SP^2^-hybridized ^◊^O) is even higher in energy than benzoanthracene (SP^2^- and SP-hybridized ^◊^O) because the former has a bad atomic Lewis structure with large steric hindrance of two closed hydrogen atoms. Compared to coronene (**5**), corannulene (**6**) is strained in both atomic and superatomic Lewis structures and thus is relatively less stable. C_60_ (**7**) has closed shells in both atomic and superatomic Lewis structures, resulting in very high stability/aromaticity.

## CONCLUSION

In summary, we proposed a unified superatomic-molecule theory to describe the local character of conjugated π electrons in PCHs, fullerenes and 2D periodic materials. Evidence by AdNDP chemical bonding frameworks and NICS(1) contour planes show that π electrons are not freely delocalized and each benzenoid superatom wants to reach the sextet rule of benzene. Electron pairs are localized in one superatom (superatomic LPs) or shared by two superatoms (superatomic bonds) and thus a molecule-like closed shell is satisfied, resulting in local aromaticity. Such an electronic behavior of conjugated π electrons mimics the rule in classical VB theory and the super sextet rule in superatomic molecules is an analogy of the octet rule in simple molecules. Benzenoid superatoms with three, four, five and six π electrons are symbolized as ^◊^N, ^◊^O, ^◊^F and ^◊^Ne, respectively. In this way, locally aromatic molecules are represented by superatomic Lewis structures and formulas, such as naphthalene (^◊^F_2_), anthracene (linear ^◊^O^◊^F_2_), phenanthrene (bent ^◊^O^◊^F_2_), triphenylene (^◊^N^◊^F_3_), coronene (cyclic ^◊^O_6_), corannulene (cyclic ^◊^O_5_) and C_60_ (^◊^N_20_ cage). Moreover, two 2D superatomic crystals, C_18_H_6_ (^◊^N_2_^◊^O_3_) and C_54_H_18_ (^◊^N_6_^◊^O_9_), are predicted, which have fairly large band gaps (∼1.8 eV) similar to covalent crystals. Superatomic-molecule theory helps us to build a reasonable chemical insight into the nature of local aromaticity, which can be qualitatively evaluated by the chemical intuition given by superatomic Lewis structures. It is really a big family for compounds/materials with conjugated π electrons. Here we do not attempt to solve everything in this single paper, but the typical examples presented in this work have given us enough confidence to believe that the superatomic-molecule theory can be a generalized language for the local aromaticity in π-conjugated systems.

## COMPUTATIONAL METHODS AND DETAILS

As a benchmark study in the general sense, the method does not affect the overall performance of π electrons. All molecules are relaxed by density functional theory (DFT) calculations at the B3LYP/6–31G* level of theory in the Gaussian 16 package [48]. MO diagrams, HOMO–LUMO energy gaps, chemical bonding analysis, NICS values and ring currents are also given at this level of theory. The NICS value above the molecular plane for 1.0 Å, namely NICS(1), is mainly due to the contribution of π electrons, which has been the most popular measurement for π aromaticity (negative NICS values represent aromaticity) [30]. To verify the global and local aromaticity, NICS(1) contour planes were given in this study instead of only the single point value. Chemical bonding analysis was carried out using the AdNDP method [44], which combines advantages of Lewis theory and MO theory to give a straightforward view of bonding elements. The AdNDP method enables *n*c–2e delocalized bonds (1 ≤ *n* ≤ the total atom number of the molecule). Periodic 2D materials were optimized by using the projector-augmented-wave (PAW) method in the Vienna ab initio simulation package (VASP) [49]. The Perdew–Burke–Ernzerhof (PBE) density functional [50] and a kinetic energy cut-off of 450 eV for the plane-wave basis set were used. The Brillouin zones were sampled using 13 × 13 × 13 Monkhorst–Pack k-point meshes.

## DATA AVAILABILITY

All data reported in this study are available upon request by contact with the corresponding authors.

## Supplementary Material

nwac216_Supplemental_File
