# Supplementary material for: A unified superatomic-molecule theory for local aromaticity in π-conjugated systems
Source: Natl Sci Rev. 2022 Oct 14;10(3):nwac216. doi: 10.1093/nsr/nwac216 (PMC10112841; doi:10.1093/nsr/nwac216)
Supplement: nwac216_Supplemental_File [file nwac216_supplemental_file.pdf]

Supplementary Informations

A unified superatomic-molecule theory for local aromaticity in π-conjugated systems

Dan Li (李丹),[1] Jinlong Yang (杨金龙),[2*] and Longjiu Cheng (程龙玖)[1*]

[1]Key Laboratory of Structure and Functional Regulation of Hybrid Materials (Ministry of Education), Department of Chemistry, Anhui Universiry, Hefei, 230601, China.

[2]Hefei National Laboratory for Physics Sciences at the Microscale, University of Science & Technology of China, Hefei, 230026, China.

*Corresponding authors. Email: clj@ustc.edu (L.C.); jlyang@ustc.edu.cn (J.Y.)

Table of Contents

- **Computational methods**
- **Figure S1**. Orbital of various angular quantum numbers. Boxed are the orbitals stretched only in *xy*-plane.
- **Figure S2.** Superatomic Lewis structures, NICS(1) contour planes (ppm), and AdNDP bonding frameworks of $C_{18}H_{12}$ ($^{\Diamond}O_2{}^{\Diamond}F_2$). (A) chrysene, (B) benzophenanthrene, (C) benzoanthracene and (D) tetracene. Enclosed are the relative energies (in kcal/mol). E_{HL} gives the HOMO-LUMO energy gaps. ON gives the occupancy numbers (|e|).
- **Figure S3.** Superatomic Lewis structures, NICS(1) contour plane (ppm), and AdNDP bonding frameworks of $C_{48}H_{24}$ ($^{\Diamond}N_6{}^{\Diamond}F_6$). ON gives the occupancy numbers (|e|).
- **Figure S4.** Superatomic Lewis structures, NICS(1) contour planes, and AdNDP bonding frameworks of (A) di-naphthalene $C_{20}H_{12}$ ($^{\Diamond}F_2$ dimer), (B) di-anthracene $C_{28}H_{14}$ ($^{\Diamond}O^{\Diamond}F_2$ dimer) and (C) tri-naphthalene $C_{30}H_{16}$ ($^{\Diamond}F_2$ trimer). ON gives the occupancy numbers (|e|).
- **Figure S5.** (A) Superatomic-molecule growth pattern of PCHs with building blocks of anthracene and triphenylene. (B) Superatomic Lewis structure, NICS(1) contour plane, and (C) AdNDP bonding frameworks of $C_{72}H_{36}$ ($^{\Diamond}N_6{}^{\Diamond}O_6{}^{\Diamond}F_6$). (D) Growth pattern from $C_{30}H_{18}$ ($^{\Diamond}N^{\Diamond}O_2{}^{\Diamond}F_4$) to $C_{18}H_6$ 2D periodic material ($^{\Diamond}N_2{}^{\Diamond}O_3$). (E) SSAdNDP orbitals of the P_x and superatomic σ bond of $^{\Diamond}N_2{}^{\Diamond}O_3$ superatomic crystal. ON gives the occupancy numbers (|e|).
- **Figure S6.** (A) Superatomic-molecule growth pattern of PCHs with building blocks of coronene and anthracene. (B) Superatomic Lewis structure, NICS(1) contour plane (ppm), and (C) AdNDP bonding frameworks of $C_{50}H_{22}$ ($^{\Diamond}N_2{}^{\Diamond}O_{11}$). (D) Growth pattern from $C_{50}H_{22}$ ($^{\Diamond}N_2{}^{\Diamond}O_{11}$) to $C_{54}H_{18}$ 2D periodic material ($^{\Diamond}N_6{}^{\Diamond}O_9$). ON gives the occupancy numbers (|e|).
- **Figure S7.** Calculated phonon dispersion curves of (A) $^{\Diamond}N_2{}^{\Diamond}O_3$ superatomic crystal (2D $C_{18}H_6$) and, (B) $^{\Diamond}N_6{}^{\Diamond}O_9$ superatomic crystal (2D $C_{54}H_{18}$).
- **Figure S8.** ACID plots of the π contribution of the studied PCHs at isosurface values of 0.035.
- **Figure S9.** ACID plots (isosurface value: 0.035) of the π contribution of **5**, coronene ($^{\Diamond}O_6$), **6**, corannulene ($^{\Diamond}O_5$), and **7**, C_{60} ($^{\Diamond}N_{20}$). The paratropic and diatropic ring currents are labeled in blue and red arrows, respectively.
- **Figure S10.** Structures, NICS(1) contour planes (ppm), and ACID plots (isosurface value: 0.035) of the π contribution of (A) $C_{48}H_{24}$ ($^{\Diamond}O_{12}$), (B) $C_{156}H_{60}$ ($^{\Diamond}N_{12}{}^{\Diamond}O_{30}$), and (C) $C_{90}H_{30}$. The paratropic and diatropic ring currents are labeled in blue and red arrows, respectively.

Computational methods

Chemical bonding analysis of periodic materials was performed using the Solid State Adaptive Natural Density Partitioning (SSAdNDP) method [1]. The anisotropy of the current-induced density (ACID) ring current was given by ACID 2.0 [2,3].

References

S1. Galeev T R, Dunnington B D, and Schmidt J R*, et al.* Solid state adaptive natural density partitioning: a tool for deciphering multi-center bonding in periodic systems. *Phys. Chem. Chem. Phys.*, 2013; **15**, 5022-9.

S2. Herges R and Geuenich D. Delocalization of Electrons in Molecules. *The Journal of Physical Chemistry A*, 2001; **105**, 3214-20.

S3. Geuenich D, Hess K, and Köhler F*, et al.* Anisotropy of the Induced Current Density (ACID), a General Method To Quantify and Visualize Electronic Delocalization. *Chemical Reviews*, 2005; **105**, 3758-72.

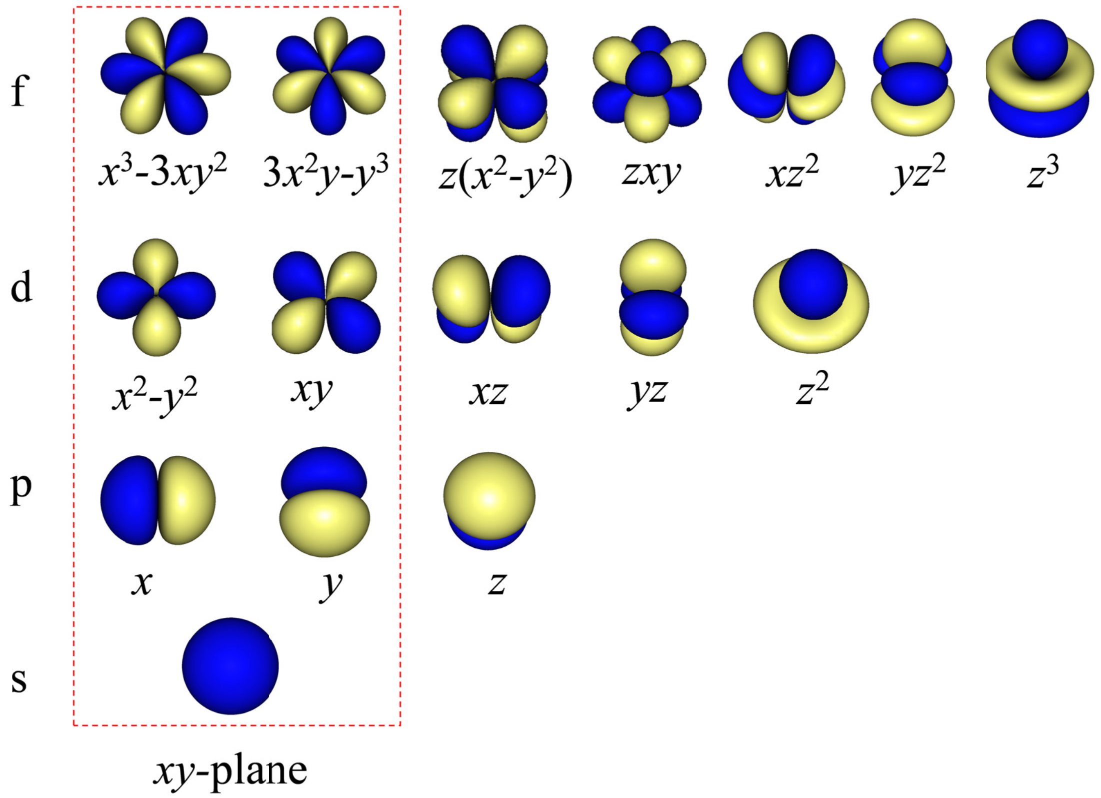

Figure S1. Orbital of various angular quantum numbers. Boxed are the orbitals stretched only in *xy*-plane.

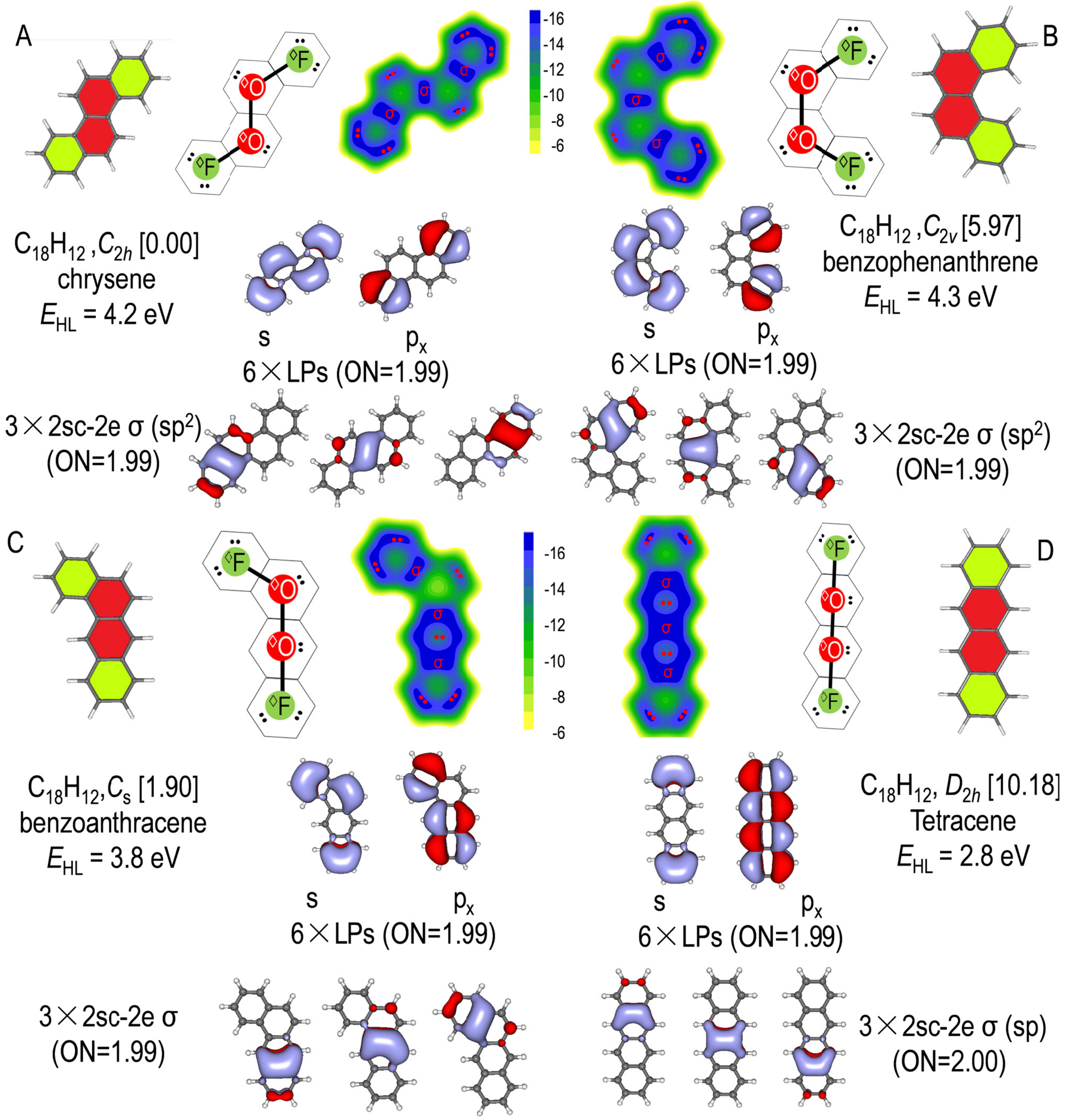

Figure S2. Superatomic Lewis structures, NICS(1) contour planes (ppm), and AdNDP bonding frameworks of $C_{18}H_{12}$ ($^{\Diamond}O_2{}^{\Diamond}F_2$). (A) chrysene, (B) benzophenanthrene, (C) benzoanthracene and (D) tetracene. Enclosed are the relative energies (in kcal/mol). E_{HL} gives the HOMO-LUMO energy gaps. ON gives the occupancy numbers (|e|).

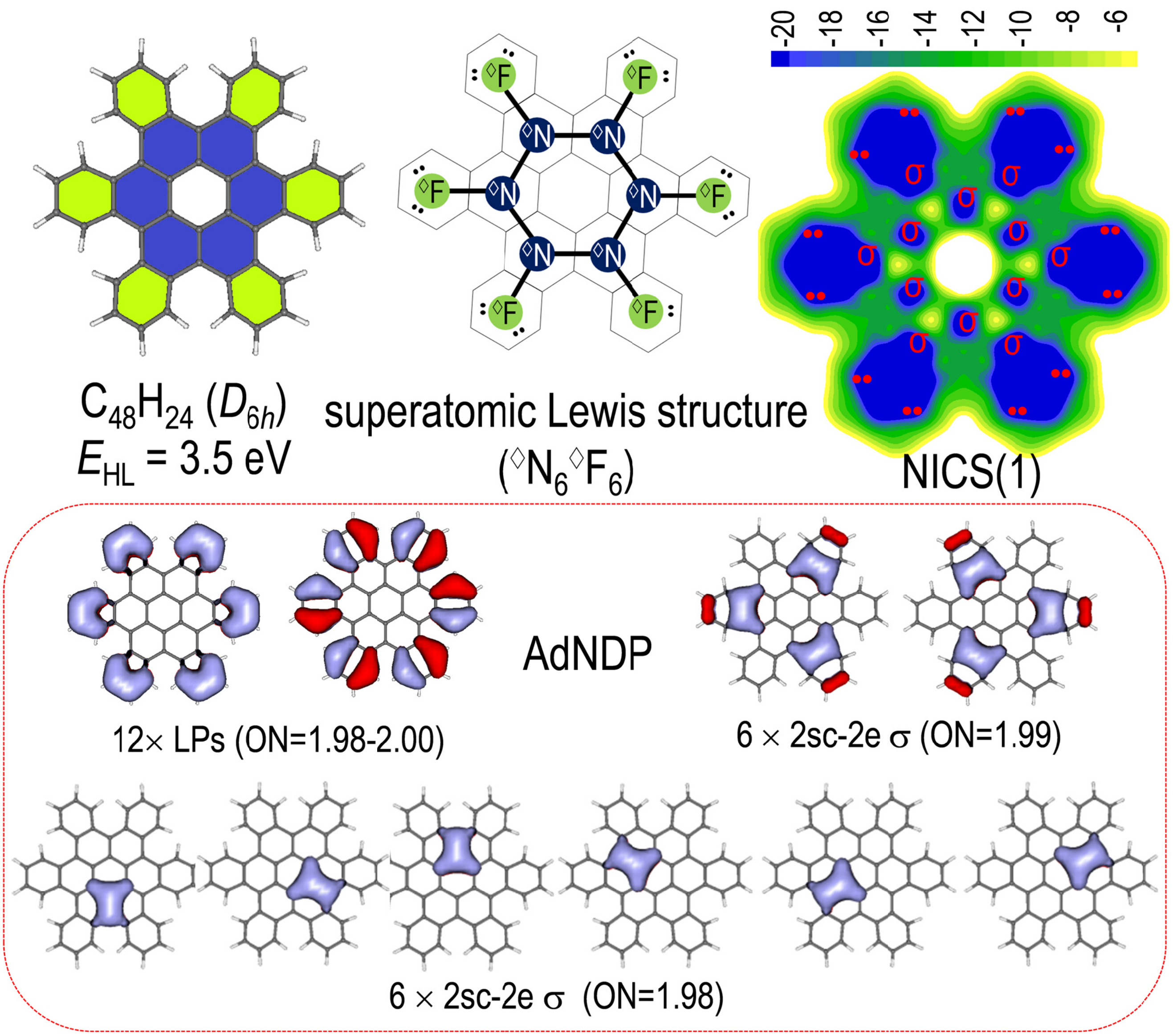

Figure S3. Superatomic Lewis structures, NICS(1) contour plane (ppm), and AdNDP bonding frameworks of $C_{48}H_{24}$ ($^{\Diamond}N_6{}^{\Diamond}F_6$). ON gives the occupancy numbers (|e|).

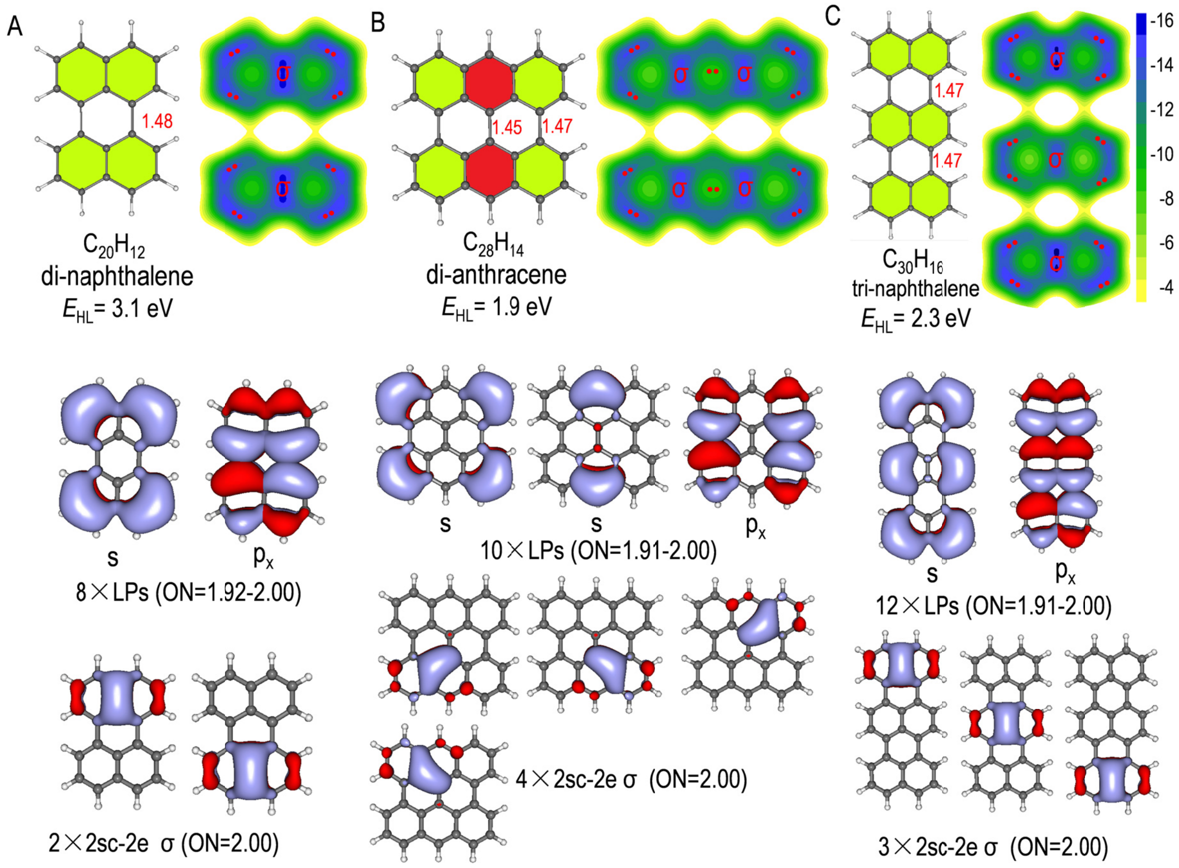

Figure S4. Superatomic Lewis structures, NICS(1) contour planes, and AdNDP bonding frameworks of (A) di-naphthalene $C_{20}H_{12}$ ($^{\lozenge}F_2$ dimer), (B) di-anthracene $C_{28}H_{14}$ ($^{\lozenge}O^{\lozenge}F_2$ dimer) and (C) tri-naphthalene $C_{30}H_{16}$ ($^{\lozenge}F_2$ trimer). ON gives the occupancy numbers (|e|).

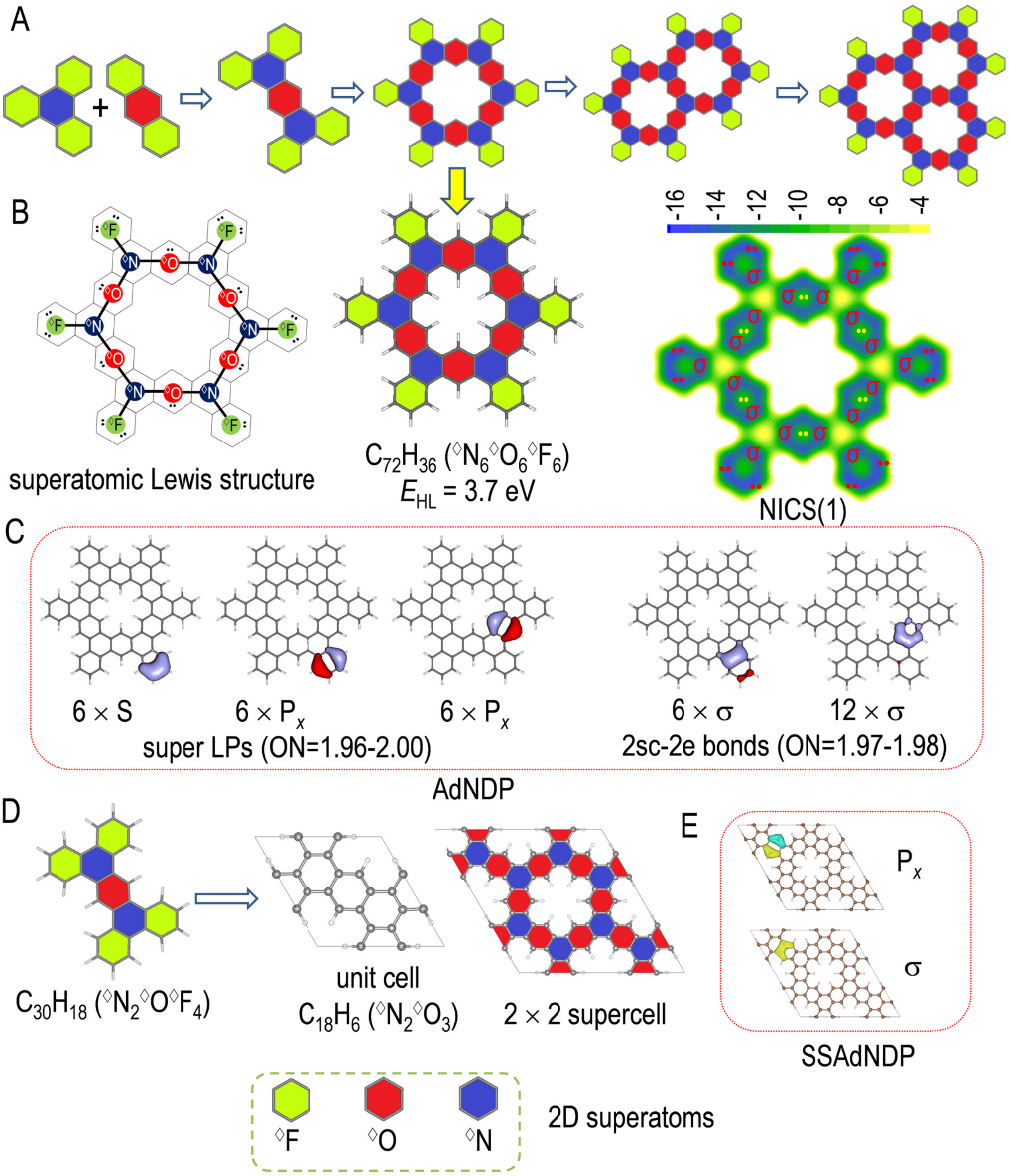

Figure S5. (A) Superatomic-molecule growth pattern of PCHs with building blocks of anthracene and triphenylene. (B) Superatomic Lewis structure, NICS(1) contour plane, and (C) AdNDP bonding frameworks of $C_{72}H_{36}$ ($^{\lozenge}N_6{}^{\lozenge}O_6{}^{\lozenge}F_6$). (D) Growth pattern from $C_{30}H_{18}$ ($^{\lozenge}N^{\lozenge}O_2{}^{\lozenge}F_4$) to $C_{18}H_6$ 2D periodic material ($^{\lozenge}N_2{}^{\lozenge}O_3$). (E) SSAdNDP orbitals of the P_x and superatomic σ bond of $^{\lozenge}N_2{}^{\lozenge}O_3$ superatomic crystal. ON gives the occupancy numbers (|e|).

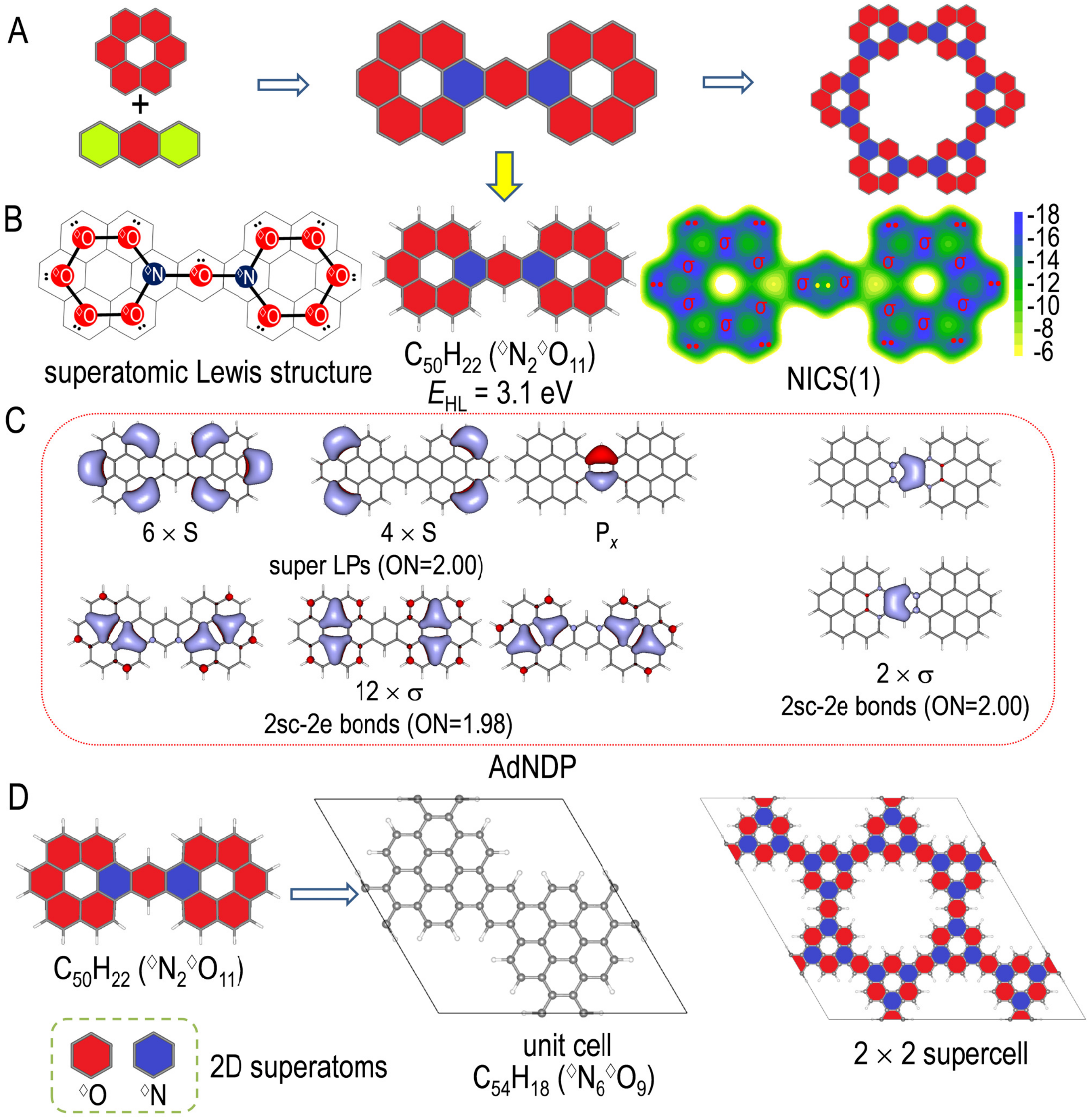

Figure S6. (A) Superatomic-molecule growth pattern of PCHs with building blocks of coronene and anthracene. (B) Superatomic Lewis structure, NICS(1) contour plane (ppm), and (C) AdNDP bonding frameworks of $C_{50}H_{22}$ ($^{\lozenge}N_2{}^{\lozenge}O_{11}$). (D) Growth pattern from $C_{50}H_{22}$ ($^{\lozenge}N_2{}^{\lozenge}O_{11}$) to $C_{54}H_{18}$ 2D periodic material ($^{\lozenge}N_6{}^{\lozenge}O_9$). ON gives the occupancy numbers (|e|).

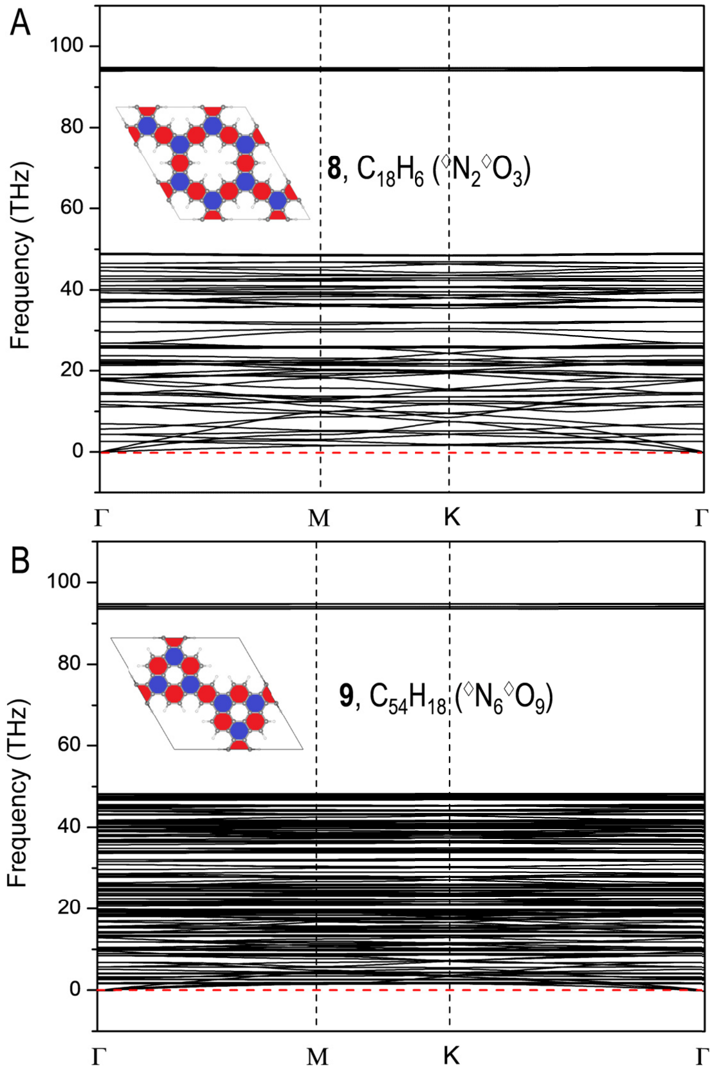

Figure S7. Calculated phonon dispersion curves of (A) $^{\Diamond}N_2{}^{\Diamond}O_3$ superatomic crystal (2D $C_{18}H_6$) and, (B) $^{\Diamond}N_6{}^{\Diamond}O_9$ superatomic crystal (2D $C_{54}H_{18}$).

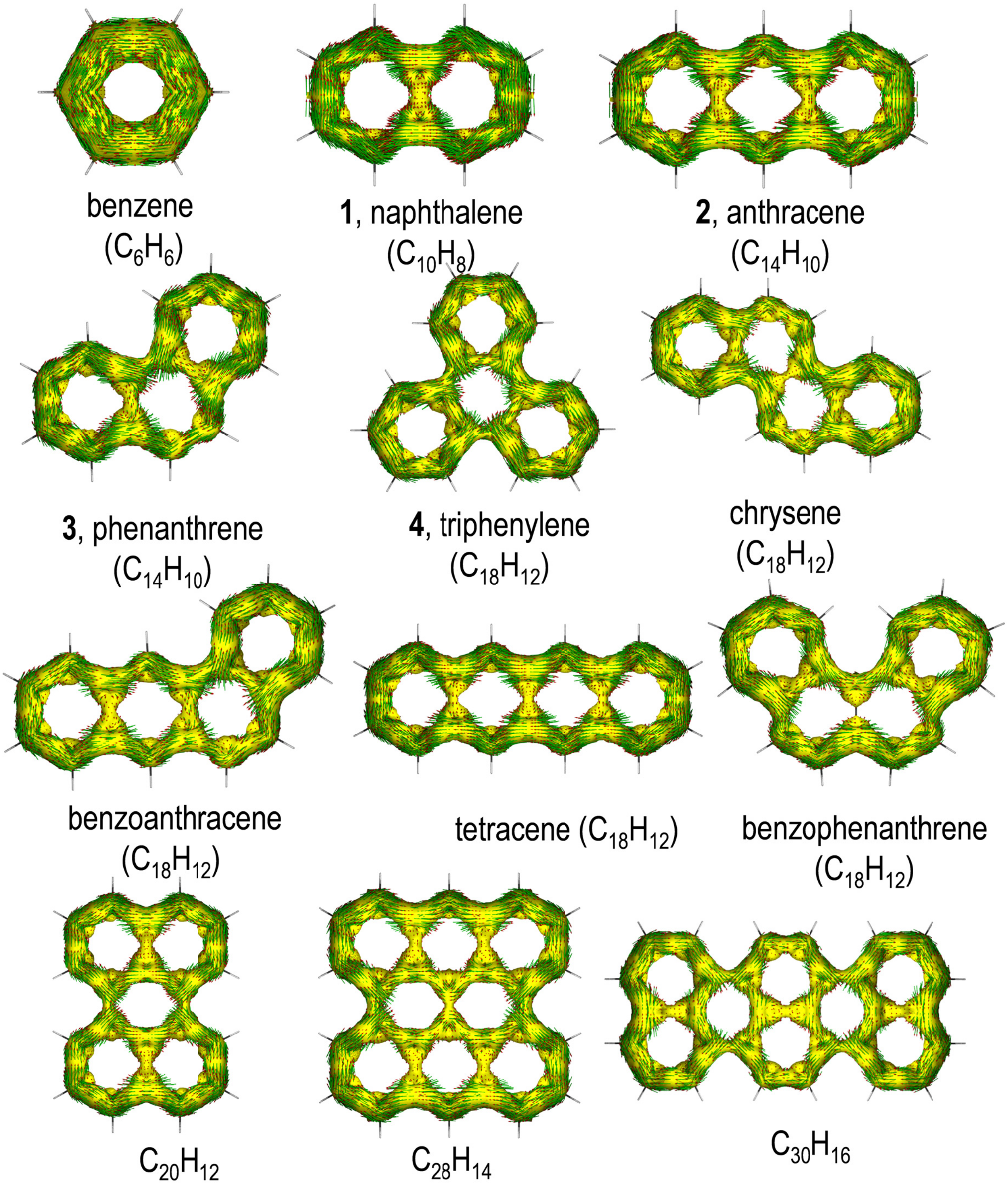

Figure S8. ACID plots of the π contribution of the studied PCHs at isosurface values of 0.035.

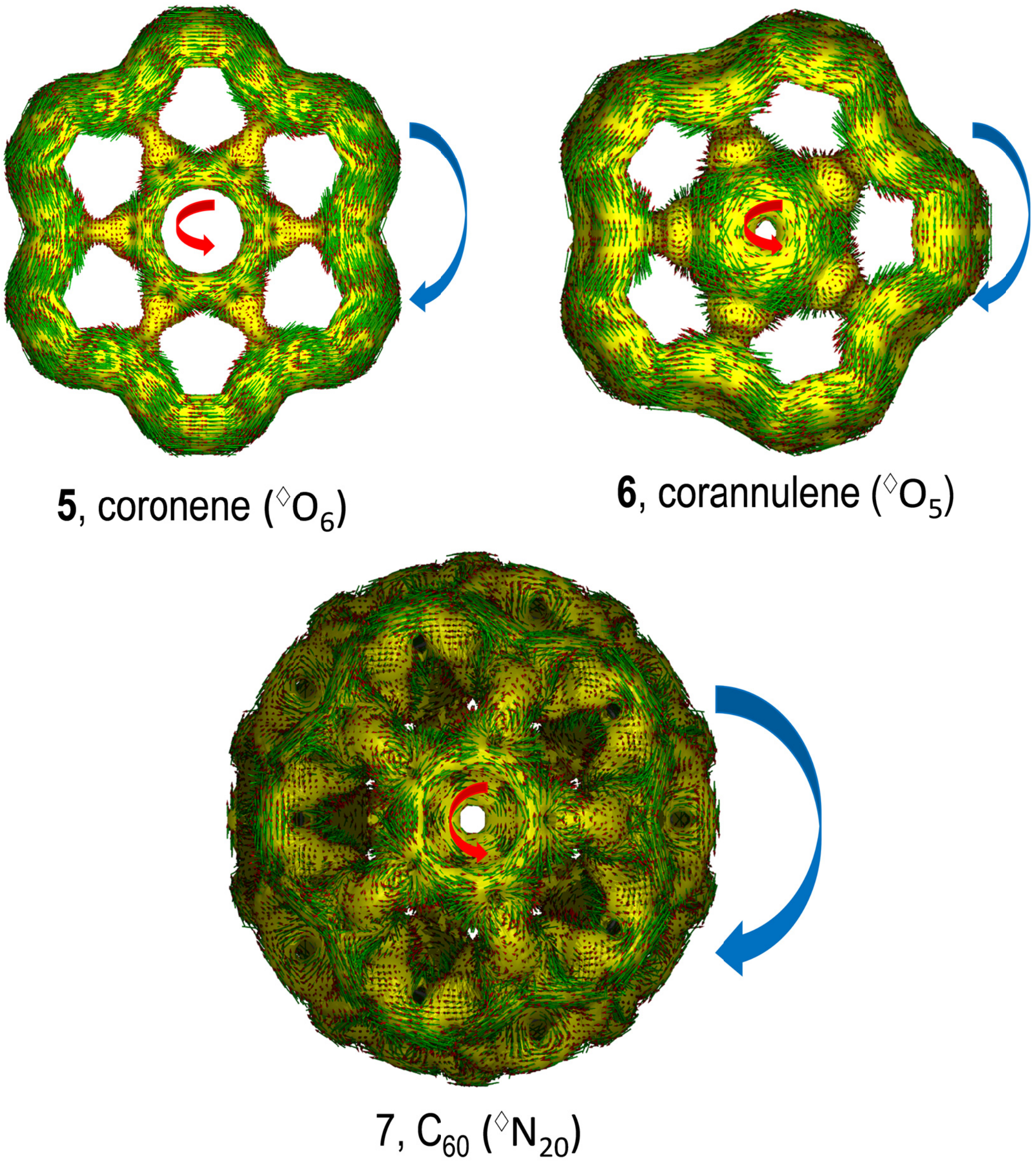

Figure S9. ACID plots (isosurface value: 0.035) of the π contribution of **5**, coronene (${}^{\lozenge}O_6$), **6**, corannulene (${}^{\lozenge}O_5$), and **7**, C_{60} (${}^{\lozenge}N_{20}$). The paratropic and diatropic ring currents are labeled in blue and red arrows, respectively.

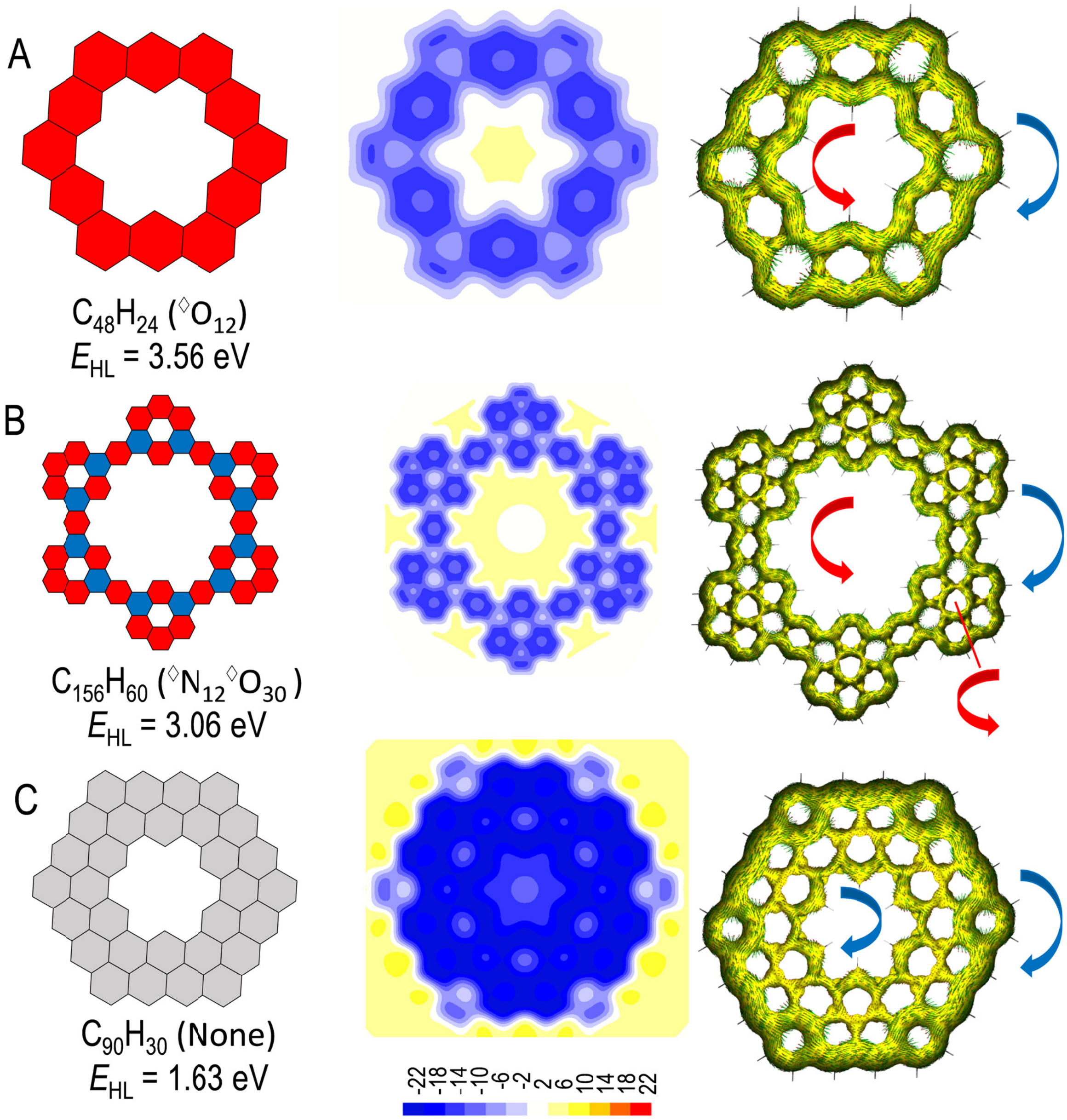

Figure S10. Structures, NICS(1) contour planes (ppm), and ACID plots (isosurface value: 0.035) of the π contribution of (A) $C_{48}H_{24}$ ($^{\lozenge}O_{12}$), (B) $C_{156}H_{60}$ ($^{\lozenge}N_{12}{}^{\lozenge}O_{30}$), and (C) $C_{90}H_{30}$. The paratropic and diatropic ring currents are labeled in blue and red arrows, respectively.
